# Estradiol to progesterone ratio is not a predictor of oocyte maturity at time of ovulation trigger

**DOI:** 10.1007/s10815-022-02491-3

**Published:** 2022-06-07

**Authors:** Marisa Berger, Hency Patel, Richard Buyalos, Gary Hubert, Chumin Wang, Mousa Shamonki, Molly Quinn

**Affiliations:** 1 Obstetrics and Gynecology, University of California Los Angeles, Los Angeles, CA USA; 2 Fertility and Surgical Associates of California, Thousand Oaks, CA USA; 3 University of Southern California, Los Angeles, CA USA

**Keywords:** Estradiol to progesterone ratio, Ovulation induction, Oocyte maturity rate, Live birth rate

## Abstract

**Purpose:**

To evaluate the relationship between progesterone and oocyte maturity rate via estradiol to progesterone ratio (E/P) at the time of ovulatory trigger.

**Methods:**

This is a retrospective cohort study of first autologous IVF cycles from January to December 2018 from a private practice fertility center. Serum estradiol and progesterone levels were measured on the day of ovulatory trigger. E/P was calculated to control for degree of response. Embryos were cultured to the blastocyst stage for trophectoderm biopsy. Preimplantation genetic testing for aneuploidy (PGT-A) was performed using next-generation sequencing (NGS). Oocyte retrieval rate (oocytes retrieved/follicles ≥ 13 mm), maturity rate (MII/oocytes retrieved), and euploid rate (euploid/total biopsied embryos) were calculated. Clinical pregnancy, ongoing pregnancy (> 10 weeks), and live births following frozen embryo transfer (FET) were examined in relation to E/P. Regression analyses were performed to analyze E/P as a categorical value (defined by quartile) on oocyte maturity.

**Results:**

Two hundred eleven women underwent controlled ovarian hyperstimulation and had steroid levels at trigger available. Mean E at trigger was 3449 ± 2040 pg/mL while mean P was 1.13 ± 0.58 ng/mL, with mean E/P of 3.36 + 2.04. There were no differences between quartiles of E/P with respect to retrieval, maturity rate, or euploid rate. Two hundred eleven IVF cycles resulted in 138 euploid frozen embryo transfers. There were no differences between quartiles of E/P with respect to clinical pregnancy, ongoing pregnancy, or live birth rate.

**Conclusion:**

E/P ratio at the time of trigger does not impact oocyte retrieval rate, maturity rate, or euploid rate. Pregnancy and live birth outcomes were also not impacted.

## Introduction

Elevated serum progesterone levels in the late follicular phase prior to ovulation trigger have been associated with decreased pregnancy rates during in vitro fertilization (IVF) with fresh embryo transfer (ET) [1–3]. Progesterone becomes elevated prior to ovulation physiologically due to the increase of theca and granulosa cells expressing enzymes that can convert cholesterol to pregnenolone and pregnenolone to progesterone [4]. Given progesterone elevation can occur in up to 38% of all stimulated cycles [5], progesterone has become a commonly collected lab test in patients undergoing controlled ovarian hyperstimulation (COH). In fresh ET, decreased pregnancy rates have been attributed to embryo-endometrial asynchrony, as the elevated progesterone can cause luteal phase endometrial gene expression and cytokine expression with putative adverse effects on the endometrium [6]. This accelerated development may be detrimental to the environment for embryo implantation, and therefore decreases pregnancy rates (PR) [7]. Multiple large retrospective studies have confirmed these findings [8, 9]. Most comprehensively, a meta-analysis containing 60,000 patients demonstrated that a progesterone level greater than 2.5 ng/mL was associated with decreased ongoing PR in fresh ET. While it has been consistently demonstrated in the literature that elevated progesterone has a detrimental effect on pregnancy rates in fresh ET, progesterone elevated to the same degree did not have an effect in 5,046 subsequent frozen ET, or in donor recipient cycles [10]. Therefore, elevated progesterone has become an indication for a freeze-all cycle for subsequent ET.

However, progesterone continues to be measured by many physicians even in planned frozen ET. Progesterone not only influences the endometrium, but its concentration can also affect developing follicles. There is growing evidence that late follicular phase progesterone may impact oocyte quality as measured by subsequent oocyte maturity or embryo quality. Elevated progesterone had negative effects on oocyte and meiotic maturation in a porcine model [11]. When 4,236 human IVF cycles analyzed the level of progesterone and its effect on top quality embryo rate, it was significantly lower in a stepwise manner as day of trigger progesterone levels increased [12]. These findings were reinforced in subsequent studies [13, 14]. However, while embryos derived from cycles with elevated late follicular phase progesterone have been associated with lower embryo quality score, this did not result in lower live birth rate (LBR) in freeze-all cycles [15]. Two recent studies, by Kofinas et al. and Hernandez-Niento et al., have eliminated the subjective aspect of embryo quality score and examined the effect of elevated progesterone on PGT-A. They found no difference in euploidy rate when progesterone was stratified by above or below 1.5 and 2.0 ng/mL, respectively [16, 17]. As a result, it remains unclear what impact, if any, an elevated late follicular phase progesterone may have on subsequent clinical outcomes in the setting of planned frozen embryo transfer.

We aimed to evaluate the impact of progesterone via estradiol to progesterone ratio (E/P) at time of ovulatory trigger on oocyte maturity as our primary outcome. We utilized E/P rather than progesterone alone to help control for degree of response from ovarian stimulation. For our secondary outcomes, we evaluated oocyte retrieval rate, euploid embryo rate, clinical pregnancy, ongoing pregnancy, and live birth rates during subsequent euploid frozen ET.

## Materials and methods

### Study design

This retrospective cohort study was approved by the UCLA Institutional Review Board. All patients who underwent autologous IVF cycles with oocyte retrievals between January 1, 2018, and December 31, 2018, at a Fertility and Surgical Associates of California were screened for inclusion in this study. Inclusion criteria dictated that women had not undergone any prior retrievals at this center, did not plan a fresh embryo transfer, and planned for PGT-A with euploid frozen ET. PGT-A was performed with next-generation sequencing (NGS). Mosaic embryos were considered aneuploid and not suitable for transfer during the study period. Cycles without a recorded progesterone or estradiol level at trigger were excluded. A total of 234 cycles were assessed, and 211 met criteria. A total of 138 euploid embryos were transferred.

### Ovarian stimulation protocols

Standard long or short GnRH agonist or GnRH antagonist protocols were used for controlled ovarian hyper stimulation. Both recombinant FSH (Follistim; Merck, Kenilworth, NJ, USA) and human menopausal gonadotropin (Menopur; Ferring Pharmaceuticals, Parsippany, NJ, USA) were used for ovarian stimulation. Patients were monitored with serial transvaginal ultrasonography to assess oocyte development. Final oocyte maturation was triggered when two follicles reached at least 18 mm in mean diameter. Serum E and P levels were measured on the day of ovulatory trigger by Immulite (Siemens Healthcare Global). Intra-assay variation for E and P was 0.2–5% and 0.1–13%, respectively. Inter-assay variation was 1.1–4.1% for E and 4.7–6.8% for P. E to P ratio in pg/mL was calculated in effort to control for degree of response. Subcutaneous GnRH agonist (leuprolide acetate 1 mg) with hCG 1000 IU together or hCG (5,000–10,000 IU) alone was used for trigger injection, followed by oocyte retrieval 35.5 h later.

### Fertilization and embryo transfer protocols

The study included both conventional insemination and intracytoplasmic sperm injection (ICSI) cases. Conventionally inseminated oocytes were co-incubated overnight in Quinn’s Fertilization with 5% HSA and a concentration of 150,000–200,000 motile sperm per mL. Once fertilization had been confirmed, the embryos were then transferred to Quinn’s Advantage Plus Cleavage media. ICSI oocytes underwent injection 5–6 h after retrieval and were cultured in Quinn’s Advantage Plus Cleavage media for 3 days. Cleavage stage embryos were next placed into Quinn’s Advantage Plus Blastocyst media (15–30 μL) for group culture. On days 5, 6, and 7, the embryos were assessed readiness for biopsy using the Schoolcraft grading system [18]. TE biopsy was performed by placing blastocysts on a holding pipette and removing 3–5 TE cells with a 20-μm biopsy pipette and cutting with via laser. After biopsy, cells went from a washing buffer to cell lysis buffer. These tubes were cryopreserved at − 20 °C and sent for testing via next-generation sequencing (NGS) at one of 3 commercially available laboratories [19, 20].

Euploid frozen embryo transfers were performed in a subsequent natural (timed by endogenous LH surge) or controlled cycle (utilizing synthetic endometrial preparation with oral estradiol and intramuscular progesterone). Clinical data including patient age, partner age, infertility diagnosis, number of blastocysts, embryo vitrification day, and FET protocol were collected.

### Outcome measures

Our primary outcome was oocyte maturity rate (MII/total oocytes retrieved).

Secondary outcomes for all cycles were oocyte retrieval rate (oocytes retrieved/number of follicles ≥ 13 mm at time of ovulatory trigger), and euploid embryo rate (euploid/ total biopsied embryos). Mosaic embryos were considered aneuploid for the purpose of this study. For cycles that underwent frozen embryo transfer, our secondary outcomes also included biochemical pregnancy (positive serum quantitative bHCG), clinical pregnancy (presence of fetal cardiac activity on ultrasound), ongoing pregnancy (pregnancy that completed > 10 weeks of gestation), and live births (live birth > 24 weeks of gestation).

### Statistics

Linear regression was performed to analyze the impact of E/P as a categorical value (defined by quartile) on clinical embryo outcomes. A *p*-value < 0.05 was considered statistically significant. Analysis was performed with chi-squared test, ANOVA, and *t*-test where appropriate.

## Results

### Study population data

A total of 234 women underwent their first cycle of COS at this clinic over the study period. Of 234 cycles evaluated, 23 were excluded due to having missing steroid levels. Of the remaining 211 patients, mean estradiol at trigger was 3449 ± 2040 pg/mL while mean progesterone was 1.13 ± 0.58 ng/mL for a mean E/P of 3.36 ± 2.04. A total of 124 women had at least one euploid embryo and underwent 138 frozen embryo transfers. The maximum number of embryos transferred at one time was 2.

The mean female age, mean male age, and stimulation day were comparable across the E/P quartiles*.* As the E/P ratio quartiles increased, the gonadotropin dose significantly decreased. The distribution of primary infertility diagnosis was notable for more DOR patients in quartiles 1 and 2 than in quartiles 3 and 4. Baseline characteristics are summarized in Table 1.

**Table 1:** Baseline characteristics

	Overall *N* = 211	E/P Q1 (*n* = 53)	E/P Q2 (*n* = 53)	E/P Q3 (*n* = 53)	E/P Q4 (*n* = 52)	*p*-value^a^
Age female, years	35.3 (3.5)	35.7 ± 3.3	35.3 ± 3.8	35.2 ± 3.5	34.9 ± 3.7	0.64
Age male, years	38.7 (6.0)	40.0 ± 6.7	38.5 ± 6.8	38.0 ± 5.3	38.4 ± 4.9	0.38
Primary infertility diagnosis						
DOR Other^c^ Male^d^ PCOS Recurrent pregnancy loss Tubal Unexplained	29 (13.7%) 71 (33.6%) 37 (17.5%) 15 (7.2%) 16 (7.6%) 16 (7.6%) 27 (12.8%)	12 12 7 3 5 6 8	12 19 12 2 1 2 5	2 21 9 2 6 7 6	3 19 9 8 4 1 8	0.012^b^
Stimulation days	8.8 (1.1)	8.7 ± 1.3	9.0 ± 1.0	8.0 ± 0.9	8.7 ± 1.0	0.40
Gonadotropin units	3315 (870)	3560 ± 980	3510 ± 730	3315 ± 755	2845 ± 820	< 0.0001
# of follicles 13 mm or greater at trigger	16.9 (8.6)	14.0 ± 8.6	15.2 ± 6.2	16.9 ± 7	21.8 ± 9.3	< 0.0001

^a^*t*-test unless otherwise indicated

^b^Chi-squared test

^c^Other includes endometriosis (9), fertility preservation (7), uterine factor (10), and elective/gender selection (12)

^d^Includes sperm donors (6)

### Embryology laboratory data

Follicle number, oocyte retrieval rate, oocyte maturity rate, and embryo euploid rate were evaluated in relation to E/P by quartile, as shown in Table 2. There was a significant relationship between the oocytes retrieved and E/P ratio, which increased as the quartiles increased, respectively. In regard to our primary outcome, oocyte maturity rate, no differences were found between quartiles of E/P. Similarly, there was no difference between quartiles in euploidy rates.

**Table 2:** Laboratory outcomes

	E/P Q1 (*n* = 53)	E/P Q2 (*n* = 53)	E/P Q3 (*n* = 53)	E/P Q4 (*n* = 52)	*p*-value^a^
Oocytes retrieved	15.0 ± 9.3	17.0 ± 8.0	18.6 ± 9.9	25.5 ± 13.0	< 0.0001
Maturity rate (MII/oocytes retrieved)	79.1 ± 15.6	80.4 ± 12.2	82.3 ± 13.7	81.2 ± 11.4	0.668
Euploid rate	53.6 ± 21.7	52.3 ± 21.5	47.0 ± 19.3	50.8 ± 24.8	0.438

^a^ANOVA

### Effect of E/P on transfer outcomes

A total of 124 women (53% of total participants) underwent frozen ET. A total of 138 embryos were transferred, with 14 women having 2 embryos transferred. Quartile 3 underwent the most embryo transfers (38), while quartile 4 underwent the least transfers (31). Effect of E/P ratio on transfer rate, implantation rate, and live birth by quartile of E/P are listed in Table 3. There were no differences between quartiles of E/P with respect to transfer or subsequent pregnancy outcomes.

**Table 3:** Transfer outcomes (*n* = 138)

	Q1	Q2	Q3	Q4	*p*-value
Underwent transfer	33 (62.3%)	36 (67.9%)	38 (71.7%)	31 (59.2%)	0.556^a^
Implantation rate	86.2 ± 35.1%	68.8 ± 45.3%	82.9 ± 38.2%	78.6 ± 41.8%	0.350^b^
Live birth rate	72.4 ± 42.5%	65.6 ± 48.3	54.3 ± 40.4%	53.6 ± 50.8%	0.370^b^

^a^Chi-square

^b^ANOVA

## Discussion

Whereas the deleterious effect of elevated progesterone on fresh embryo transfer cycles is established, the impact on frozen cycles has been the topic of debate. This question continues to be clinically relevant, as serum progesterone is routinely collected in ovarian stimulation, even for planned frozen cycles, but there are no guidelines on how to interpret or use this data. More recent studies have interpreted elevated progesterone as a cause for suboptimal oocyte competence and have attempted to define a threshold that indicates an inferior outcome [14, 17, 18, 21].

To our knowledge, this is the first study to attempt to control for response to ovarian stimulation by using a ratio of estradiol to progesterone in frozen embryo transfer. Estradiol and progesterone both increase with the level of response to ovarian stimulation [22]. Increasing estradiol correlates with increasing follicle number with the attendant increased development of granulosa cells [23]. However, one hypothesis states that progesterone may increase at a greater rate than estradiol in the late follicular phase during ovarian stimulation because mechanisms to prevent premature luteinization are disrupted [24]. For this reason, utility of a ratio of estradiol to progesterone instead of progesterone alone has been examined with fresh ET, and similar negative correlations between elevated E/P ratio and implantation rate, PR, and LBR have persisted [25–27]. However, the use of a ratio has previously not been investigated in frozen ET.

By utilizing E/P ratio, we sought to reflect the relationship of progesterone level more accurately with follicle number and oocyte maturity. Progesterone elevation has been interpreted as a reflection of the maturity of a cohort of follicles, but there is no evidence that this is true. Progesterone elevation has also been interpreted as a contributing factor to abnormal oocyte development. In fact, our data demonstrated neither, as no difference was found in oocyte maturity rate or euploid rate from the lowest to highest levels of E/P by quartile. Our findings correlated with both the Kofinas and Hernandez-Niento studies, demonstrating no decrease in embryonic competence with rising E/P in a frozen embryo transfer cycle.

Our study also builds on these findings by refining our independent variable, progesterone, by adjusting for each individual’s response to ovarian stimulation by the total estradiol production. This allowed us to delineate two different categories of responders: (1) those who are poor responders, and will a low E/P, with rising progesterone levels exceeding rise in estradiol levels and (2) those who are excellent responders, who in previous studies would have been considered to have “elevated P,” but, when adjusted for their steroidogenic response with similarly elevated estradiol, have an E/P closer to the mean. For example, in a study of oocyte donation cycles, serum progesterone > 1.5 ng/mL was associated with increased mean oocytes retrieved and good quality day 3 embryos [28]. In studies that hypothesized that supraphysiologic P would be detrimental to IVF outcomes, this second group would be prone to type I error.

Similar to the Hernandez-Niento study, one of our study strengths was consistent use of PGT-A in all cycles, which allowed for genetic assessment of embryo quality rather than solely utilizing subjective morphologic assessments. An additional advantage our study is consistency, with all of our cycles occurring in the same embryology lab. The downside of being in a single center is our relatively small sample size. While we only examined 211 controlled ovarian hyperstimulation cycles, this was from 211 discrete patients with no repeat cycles, and therefore our data set was not skewed by patients who required multiple stimulation cycles. Regarding our transfer and pregnancy outcomes, 138 embryos were transferred, with all patients undergoing their first transfer attempt at this clinic. As we included a convenience sample of all patients meeting criteria over the study period, we did not perform a pre-specified power analysis. Therefore, it is possible that our study is underpowered, and therefore at risk for type II error, particularly with respect to small differences in our outcome of interest. However, despite our smaller sample size, it is reassuring that the data for our primary and secondary outcomes are consistent with the Hernandez-Niento paper, which was powered to evaluate the effect of progesterone levels in frozen embryo transfer on euploidy, blastulation, and implantation rates.

Another limitation of all studies evaluating the effect of preovulatory progesterone on IVF outcomes is the precision of progesterone assay testing. Our embryology lab utilized Immulite, which has an intra and inter assay variation of 0.1–13% and 4.7–6.8% for progesterone, and 0.2–5% and 1.1–4.1% for estradiol, respectively. Preovulatory progesterone levels are physiologically low, typically in the range from 0 to 3 ng/mL. Therefore, small variations in the detected progesterone level could have resulted in erroneous categorization into a higher or lower quartile. This limitation was also mentioned in the discussion of the Kofinas and Hernandez-Niento studies, which further questions the clinical utility of collecting preovulatory progesterone in frozen embryo transfer cycles.

In conclusion, this study demonstrates that late follicular E/P ratio during ovarian stimulation may not be a useful indicator of oocyte maturity, and therefore should not be used as a factor when guiding clinical decision to trigger ovulation. Elevated P alone or E/P ratio has not demonstrated an ability to predict oocyte quality or embryo euploidy. The study also confirms that elevated progesterone does not affect cycle outcomes, from biochemical pregnancy to live births in subsequent frozen embryo transfer cycles. Therefore, these data, supported with data from other studies, suggest that collecting a late follicular progesterone level during ovarian stimulation is not indicated for patients who are undergoing planned frozen embryo transfers. We suggest that for planned frozen embryo transfers, patient resources are used judiciously and to not routinely include progesterone in pre-trigger laboratory orders, particularly in patients in which one would expect a robust response to ovulation induction.
